# Gallstone ileus developing two months after endoscopic papillary large balloon dilatation: a case report

**DOI:** 10.1186/s13256-026-06299-y

**Published:** 2026-07-02

**Authors:** Yutaka Matsuzaki, Koh Matsumoto, Sumika Oyama, Hiromi Saito, Hitomi Fujimoto, Akira Kameyama, Daisuke Murayama, Toru Igarashi, Norikazu Arakura, Kendo Kiyosawa

**Affiliations:** 1 Department of Gastroenterology, Aizawa Hospital, 2-5-1 Honjo, Matsumoto, Nagano 390-8510 Japan; 2 Department of Surgery, Aizawa Hospital, 2-5-1 Honjo, Matsumoto, Nagano 390-8510 Japan

**Keywords:** Gallstone ileus, Endoscopic retrograde cholangiopancreatography, Endoscopic papillary balloon dilatation, Endoscopic complications

## Abstract

**Background:**

Gallstone ileus is a rare clinical entity causing bowel obstruction, and transpapillary migration of gallstones after endoscopic retrograde cholangiopancreatography is exceptionally uncommon. Although endoscopic papillary large balloon dilatation is an effective procedure that dilates the Vater’s papilla to remove larger size calculi, gallstone ileus following the procedure as a complication has rarely been reported in the literature.

**Case presentation:**

We present the case of an 89-year-old Japanese woman diagnosed with acute cholangitis due to choledocholithiasis, exhibiting epigastric pain, nausea and jaundice. She was treated with endoscopic procedure, including papillary large balloon dilatation, but attempts at stone extraction were unsuccessful. Two months later, the patient was readmitted with acute cholangitis, likely secondary to biliary stent occlusion; however, retrospective review of the computed tomography revealed multiple common bile duct stones as well as a large intrajejunal foreign body consistent with a stone. One day after undergoing urgent endoscopic procedure, she developed recurrent vomiting and was diagnosed with gallstone ileus due to impaction of the stone in the ileum. Following failed endoscopic disimpaction, urgent surgery was performed, and the stone was manually fragmented into several pieces and pushed distally without the need for enterotomy. She was discharged from the hospital on eighth day after the operation.

**Conclusion:**

To our knowledge, this is one of the very few cases of gallstone ileus occurring after endoscopic papillary large balloon dilatation. When extraction of a large common bile duct stone is difficult even after endoscopic papillary large balloon dilatation, additional treatment strategies should be considered, given the potential risk of delayed spontaneous passage and subsequent gallstone ileus.

## Background

Gallstone ileus is a rare clinical presentation of bowel obstruction due to impaction of a calculus in the intestinal loop. The causative large gallstone from the gall bladder or common bile duct, mostly 2–3 cm or more in diameter, enters mainly via biliary-enteric fistula, and rarely via the trans-papillary route after endoscopic sphincterotomy (EST) [1, 2]. Endoscopic papillary large balloon dilatation (EPLBD) is an effective procedure that dilates the Vater’s papilla with a balloon more than 10 mm in diameter to remove large size calculi [3]. Although gallstone ileus may occur after EPLBD, it has rarely been reported in the literature.

## Case presentation

**Fig. 1:**
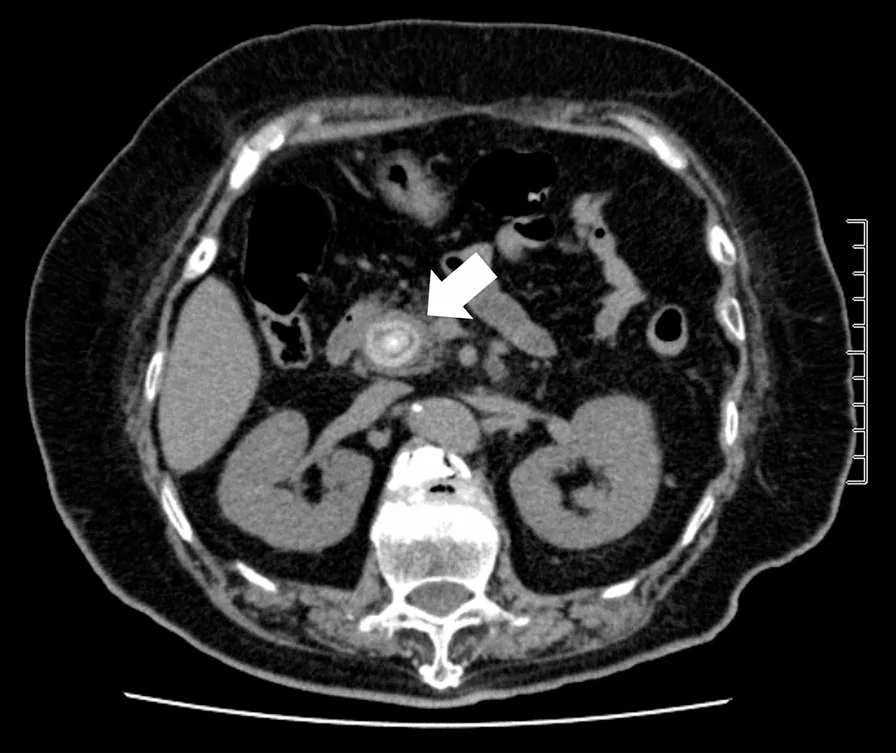
Abdominal CT scan at the first visit revealing dilatation of common bile duct and large choledocholithiasis (arrow)

We report an 89-year-old Japanese woman who presented to our emergency department with liver dysfunction and jaundice, preceded by several weeks of anorexia, nausea and epigastric pain. Her past medical history included appendectomy for appendicitis, thyroidectomy for goiter, hypertension and osteoporosis. She had no history of ERCP or hepatobiliary surgery. She did not smoke or consume alcohol. Physical examination revealed normal vital signs except for tachycardia of 130/minute. Scleral icterus and jaundice were noted, along with epigastric tenderness and tenderness to percussion over the right lower rib area. Laboratory findings showed remarkably elevated white blood cells (31.9 ⨉10^9^/L, neutrophile dominant), C-reactive protein (237.3 mg/L) and liver test abnormalities (aspartate aminotransferase 209 U/l, alanine aminotransferase 194 U/l, γ-glutamyl-transpeptidase 499 U/l) as well as jaundice (total bilirubin 165.9 µmol/L). Contrast-enhanced computed tomography (CT) revealed a common bile duct stone measuring 2–3 cm in diameter, along with biliary dilatation (Fig. 1). She was diagnosed with relatively severe acute cholangitis due to choledocholithiasis. She was immediately admitted and underwent urgent ERCP after initiation of antibiotics. Endoscopic cholangiogram (ERC) showed a large stone in the lower common bile duct measuring 30 × 20 mm, with additional smaller stones also suspected. Hence extraction of the stone was estimated to be difficult even after middle-incision EST following EPLBD of 15 mm diameter, biliary stenting (7Fr, 10 cm, double pigtail, plastic) was performed. The post-procedural course was uneventful, and the patient was discharged 13 days later with the plastic biliary stent in-place and ursodeoxycolic acid medication. In the event of recurrent cholangitis, the management plan was to perform biliary stent exchange or refer the patient to a specialized center capable of advanced endoscopic treatment, including electrohydraulic lithotripsy (EHL).

**Fig. 2:**
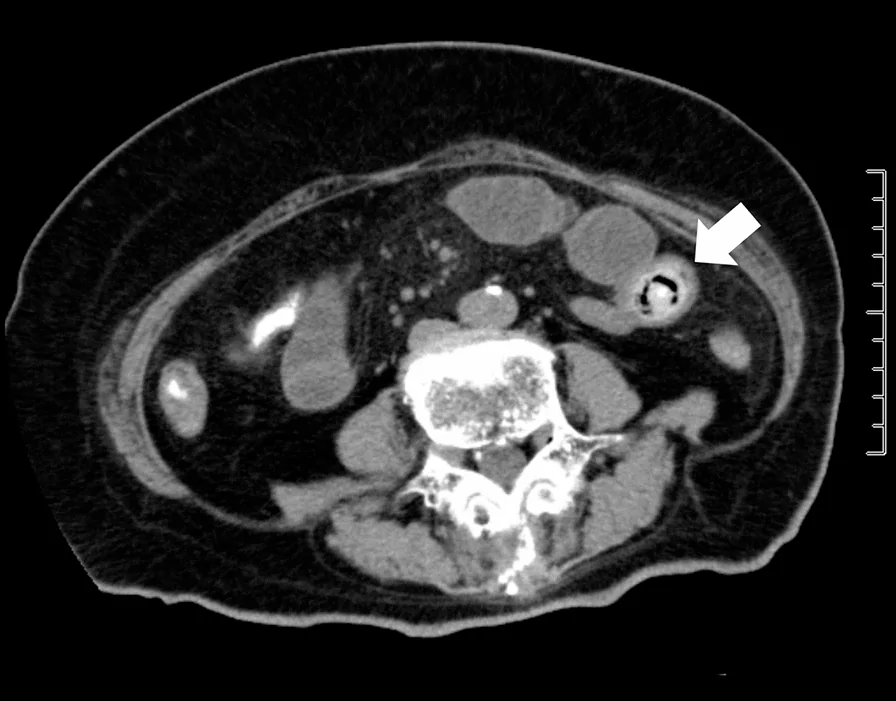
Abdominal CT scan two days after the second ERCP revealing ileus with caliber change at foreign body in the ileum (arrow)

**Fig. 3:**
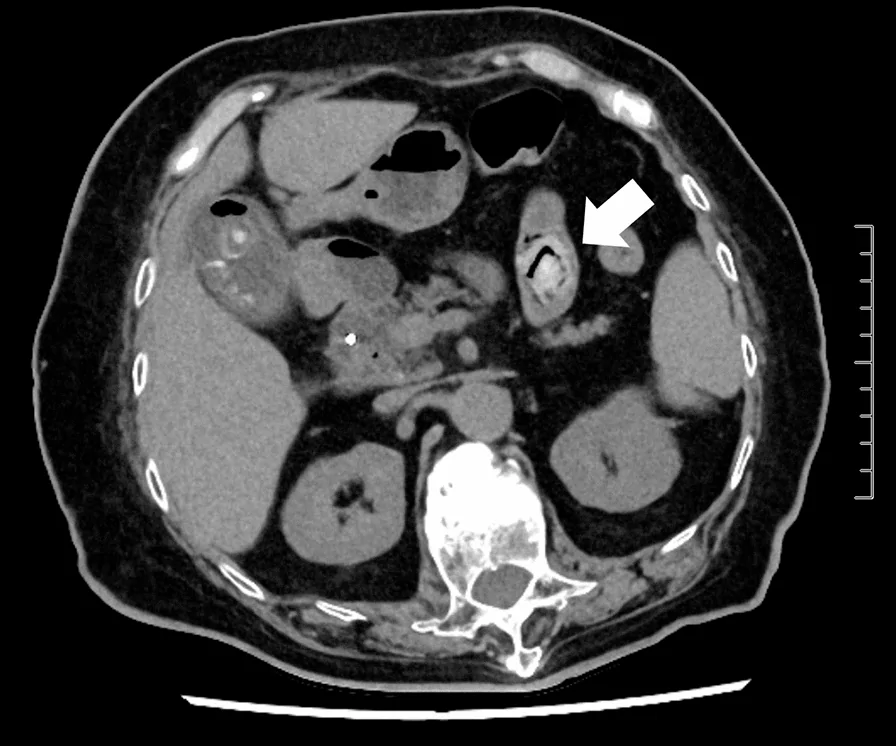
Abdominal CT scan at the second visit BEFORE ERCP revealing foreign body in the upper jejunum (arrow)

**Fig. 4:**
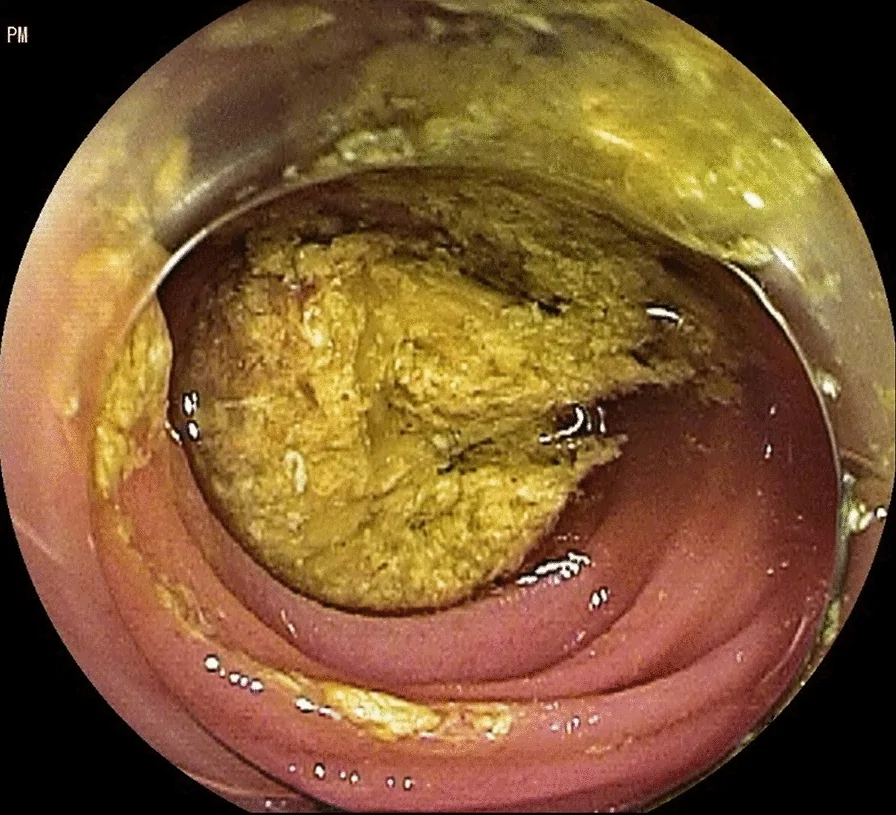
Double-balloon endoscopic appearance of the impacted gallstone in the ileum

**Fig. 5:**
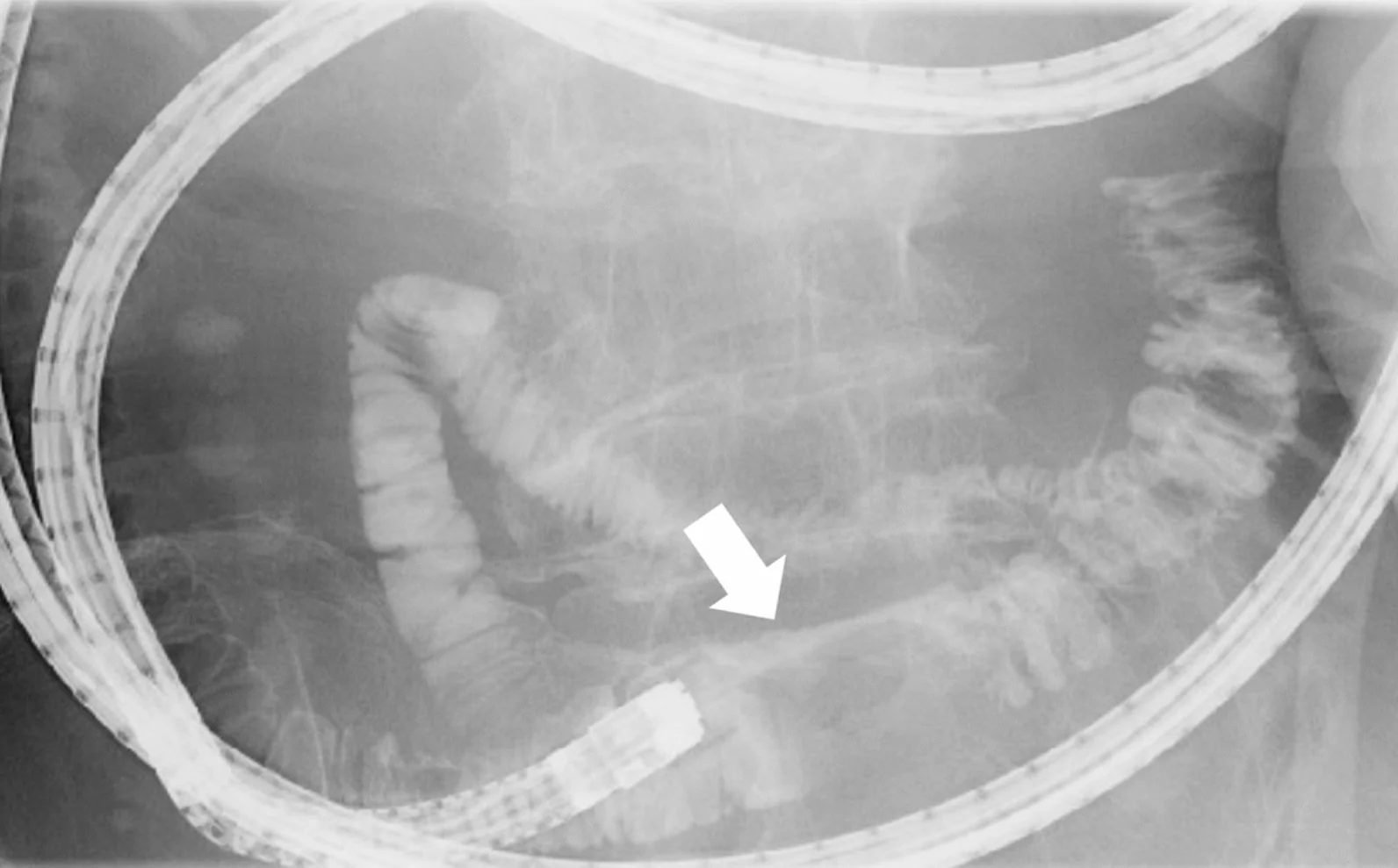
Endoscopic contrast study with gastrografin revealed a radiolucent gallstone impacted in the ileum (arrow)

Two months later, the patient developed epigastric pain and nausea again. The next day, she was referred to our hospital for jaundice. Vital signs were body temperature of 37.0℃, blood pressure of 120/97 mmHg, heart rate of 130/minute. Physical examination showed icteric conjunctiva and skin, abdominal tenderness in the epigastric and upper-right regions. Laboratory findings revealed elevated white blood cells (15.7 ⨉10^9^/L,) and C-reactive protein (68.4 mg/dl) as well as liver enzyme (aspartate aminotransferase 157 U/l, alanine aminotransferase 75 U/l, γ-glutamyl-transpeptidase 400 U/l, total bilillubin 68.4 µmol/L). Contrast-enhanced CT demonstrated an intact plastic biliary stent, pneumobilia and stones in the common bile duct of which characteristics had changed compared to previous imaging. She was diagnosed with acute cholangitis, likely secondary to biliary stent occlusion and common bile duct stones, and was immediately admitted for antibiotic therapy and urgent ERCP. At the second ERCP, the largest stone measured no more than 20 × 15 mm, with multiple smaller stones also present and a change in morphology noted on cholangiography. The plastic stent was removed, and lithotripsy was performed using an endoscopic mechanical lithotripsy (EML) basket device without additional EST, EPLBD, or placement of a new biliary stent. The papillary orifice appeared dilated, likely due to the prior papillary intervention. Multiple extracted stones were yellow and consistent with bilirubin stones, and no residual stones were evident in the bile duct. On the day after admission (day 1), although laboratory data showed remarkable improvement of acute cholangitis, the patient developed new symptoms of bilious vomiting, intermittent abdominal pain, and tenderness in the lower-left quadrant. On day 2, a plane CT showed dilation of small bowel loops up to a point in the left flank with a calcified foreign body (approximately 3 cm in diameter) with standard-caliber distal loops (Fig. 2). Retrospective view of the CT images obtained prior to second ERCP on the same day demonstrated the same foreign body in the upper jejunum without evidence of bowel obstruction, indicating that the common bile duct stone had already passed spontaneously before second ERCP (Fig. 3). No biliary-enteric fistula was identified during either of the two previous ERCP procedures or during the balloon-assisted enteroscopy described below. These findings suggest that the gallstone ileus was caused by a stone that had spontaneously passed through Vater’s papilla two months after EPLBD. Conservative management, including nasogastric decompression and fluid resuscitation, was attempted. However, persistent high nasogastric drainage and decreased urine output suggested difficulty with continued conservative treatment. On day 3, oral double-balloon enteroscopy reached the impacted gallstone, but endoscopic removal was unsuccessful (Fig. 4, 5). Therefore, tattooing of the oral side of the intestinal wall was performed to facilitate subsequent surgical localization. Regarding the management of gallstone ileus, conservative approaches, including dissolution therapy and watchful waiting, were considered. However, as the impaction had not resolved despite more than 24 h having elapsed since onset of the bowel obstruction, and a review of the literature indicated that successful conservative management is uncommon and that no established non-surgical treatment exists, emergency surgery was selected. During laparotomy on the same day, a 3 cm gallstone was palpated through the intestinal wall in the ileum adjacent to the intestinal tattoo. The stone was crushed manually in several pieces without enterotomy. Her post-operative recovery was uncomplicated and she was discharged eight days after surgery.

## Discussion and conclusions

Gallstone ileus is an uncommon bowel obstruction caused by an impacted gallstone from the gallbladder or bile duct, which accounts for 0.1% or less of ileus cases overall [2]. Generally, causative gallstones are 3 cm or larger, but cases with 2 cm or smaller stones have been reported depending on the patient’s background (intestinal stricture, post-operative adhesion, natural caliber narrowing like an ileocecal valve) [1]. The major route by which gallstones pass into the intestinal tract is a bilioenteric fistula (especially a cholecystoduodenal fistula). Gallstone ileus following ERCP, the stone passing via Vater’s papilla, is exceedingly rare due to the relatively smaller outlet diameter compared to bilioenteric fistula. We defined “post-ERCP gallstone ileus” as mechanical bowel obstruction caused by impaction of a gallstone presumed to have passed transpapillary after ERCP, excluding cases associated with biliary-enteric fistulae, regardless of the presence or absence of underlying gastrointestinal stenosis or other intestinal diseases. A PubMed literature search from database inception to April 2026 using the following search terms: (“gallstone ileus” OR “bowel obstruction”) AND (“endoscopic retrograde cholangiopancreatography” OR “endoscopic sphincterotomy” OR “ERCP” OR “papillary balloon dilatation” OR “papillary large balloon dilatation”) identified 20 reported cases of post-ERCP gallstone ileus without bilioenteric fistula, including the present case (Table 1) [4–22]. Only English-language full-text articles with sufficient clinical details were included. Cases of Bouveret syndrome were excluded because the underlying mechanism differs from post-ERCP gallstone ileus, as Bouveret syndrome is typically associated with spontaneous stone migration through a bilioenteric fistula causing proximal gastrointestinal obstruction. Our review of the reported cases revealed that most patients were elderly (18/20 were aged 65 years old or older) and had predisposing conditions potentially associated with intestinal narrowing, including prior abdominal surgery or abdominal radiotherapy (15/20). Among the endoscopic procedures that triggered the ejection of a gallstone from bile duct into the intestine, all were EST except for the present case of EPLBD. The period between ERCP and the onset of gallstone ileus varied considerably from daily to monthly. More than half of the impacted gallstones (11/20) were 3 cm or larger in diameter, although smaller gallstones were also associated with gallstone ileus. Regarding treatment, enterotomy was performed in more than half of the cases (11/20). However, several patients were successfully treated without enterotomy, including conservative management, endoscopic lithotripsy, and surgical manipulation of the impacted gallstone without enterotomy, as in the present case. To the best of our knowledge, this is the first reported case of gallstone ileus occurring after EPLBD. EPLBD is an effective technique for dilating the major duodenal papilla and extracting relatively large bile duct stones [3]. According to the Japanese clinical practice guidelines for EPLBD, adverse events such as bleeding, perforation, pancreatitis, and cholangitis/cholecystitis are listed; however, gallstone ileus is not mentioned [23]. Although gallstone ileus is a very rare complication after ERCP, the present case suggests that it can occur following papillary interventions including EST but also EPLBD. In cases where large bile duct stones cannot be completely extracted despite EPLBD, additional strategies to achieve or confirm duct clearance should be considered, such as stone fragmentation techniques (including EML or EHL) or scheduled second-look cholangiography. In particular, retained large stones may migrate distally through a persistently dilated papilla and subsequently cause bowel obstruction, even when the stone diameter substantially exceeds the balloon size used for EPLBD, as demonstrated in the present case in which a 3 cm gallstone became impacted despite an EPLBD balloon diameter of only 15 mm. This case underscores the importance of recognizing incomplete bile duct stone clearance after EPLBD as a potential risk factor for delayed gallstone ileus. Careful post-procedural follow-up may therefore be warranted, especially in high-risk patients with a history of large choledocholithiasis or predisposing conditions associated with intestinal narrowing. However, as this is a single case report with limited follow-up, no definitive conclusions regarding risk stratification or preventive strategies can be drawn, and evidence supporting specific interventions to prevent gallstone ileus remains lacking. This case underscores the importance of recognizing that gallstone ileus can occur as a delayed complication after EPLBD, even when the stone size is significantly larger than the diameter of the dilation balloon. Clinicians should be aware that large bile duct stones may migrate into the intestine and cause obstruction over time, despite apparent initial resolution after ERCP. Careful follow-up may therefore be considered in selected patients with large choledocholithiasis or technically difficult stone extraction. However, as this is a single case with limited follow-up, further studies and accumulation of similar cases are needed to clarify the mechanisms and risk factors of this rare complication.

**Table 1:** Summary of reported cases of gallstone ileus following ERCP

Ref	Age	Sex	Predisposing factors	Endoscopic procedure	Time from ERCP to onset of gallstone ileus	Diameter of stone	Site of impaction	Treatment
4	60	F	Abdominal surgery	EST	3 days	3.5 cm	Jejunum	Enterotomy
5	100	F	Abdominal surgery	EST	1 day	1.9 cm	Terminal ileum	Manual propulsion
6	79	M	Abdominal surgery	EST, ENBD	3 days	2.8 cm	Terminal ileum	Enterotomy
7	79	F	Abdominal surgery	EST	1 day	2 cm	Intestine (unk)	Non (spontaneous passage)
8	72	F	Abdominal radiotherapy	EST	3 weeks	3 cm	Intestine (unk)	Enterotomy
9	81	F	Non	EST, EBS	2 weeks	3 cm	Jejunum	Non
10	71	M	N/A	EST	2 months	4 cm	Jejunum	Enterotomy
11	84	F	Abdominal radiotherapy	EST	5 days	2 cm	Terminal ileum	Enterotomy
12	84	F	N/A	EST	2 weeks	NA	Ileum	Enterotomy
13	67	F	Abdominal surgery, Crohn’s disease	EST	N/A	3 cm	Terminal ileum	Enterotomy
14	86	M	Abdominal surgery	EST	3 months	2.5 cm	Terminal ileum	Non
15	67	F	Abdominal surgery	EST	4 months	3 cm	Jejunum	Enterotomy
16	69	M	Non	EST	2 days	2.3 cm	Terminal ileum	Enterotomy
17	85	F	Non	EST	1 day	4.5 cm	Terminal ileum	Non (spontaneous passage)
18	63	F	Abdominal surgery	EST, EBS	4 days	3.9 cm	Ileum	Endoscopic lithotripsy
19	81	M	Abdominal surgery	EST	4 days	2.8 cm	Ileum	Enterotomy
20	78	F	Abdominal surgery	EST	6 months	3.5 cm	Terminal ileum	Enterotomy
21	92	M	Abdominal surgery	EST	7 months	5 cm	Terminal ileum	Enterotomy
22	88	F	Colon cancer	EST	11 days	1 cm	Sigmoid colon	Sigmoidectomy with colostomy
Present case	89	F	Abdominal surgery	EPLBD, EBS	2 months	3 cm	Ileum	Manual fragmentation

## Data Availability

Not applicable.

## Abbreviations

ERCP: Endoscopic retrograde cholangiopancreatography
EPLBD: Endoscopic papillary large balloon dilatation
EST: Endoscopic sphincterotomy
EHL: Electrohydraulic lithotripsy
EML: Endoscopic mechanical lithotripsy
CT: Computed tomography
